# Emerging Role of Dual Glucagon-Like Peptide-1 (GLP-1)/Glucose-Dependent Insulinotropic Polypeptide (GIP) Receptor Agonists in Cardiovascular Prevention

**DOI:** 10.7759/cureus.104567

**Published:** 2026-03-02

**Authors:** Nicolle Contreras Figueroa, Maynor Jose Lopez Mendoza, Asdrubal Ulloa, Jeilyn Jiron Vindas, Maria Antonieta Salazar Estrada, María Jennifer Valle Mena

**Affiliations:** 1 General Medicine, Caja Costarricense de Seguro Social (CCSS), San Jose, CRI; 2 Anesthesiology and Perioperative Medicine, Hospital de las Mujeres Dr. Adolfo Carit Eva (HOMACE), San Jose, CRI; 3 Gynecologic Oncology, Hospital de las Mujeres Dr. Adolfo Carit Eva (HOMACE), San Jose, CRI; 4 Obstetrics and Gynecology, Hospital de las Mujeres Dr. Adolfo Carit Eva (HOMACE), San Jose, CRI; 5 Emergency Medicine, Hospital Los Chiles, Los Chiles, CRI; 6 General Medicine, Área de Salud Upala, Upala, CRI

**Keywords:** cardiometabolic risk, cardiovascular prevention, endothelial function, incretin physiology, insulin sensitivity, visceral adiposity

## Abstract

Dual glucagon-like peptide-1 (GLP-1) and glucose-dependent insulinotropic polypeptide (GIP) receptor agonists have emerged as a novel therapeutic class with potential relevance for cardiovascular prevention, particularly in the context of obesity and type 2 diabetes mellitus. Incretin physiology provides the biological foundation for this approach, as GLP-1 and GIP exert complementary metabolic and vascular effects. While GLP-1 receptor agonists have demonstrated well-established reductions in major adverse cardiovascular events, GIP has regained interest due to evidence suggesting preserved vascular and anti-atherosclerotic actions despite reduced insulinotropic efficacy in diabetes.

Dual receptor agonism integrates these pathways, leading to substantial improvements in cardiometabolic risk factors. Agents such as tirzepatide induce marked and sustained weight loss, with significant reductions in visceral adiposity, a key driver of cardiovascular disease. These effects are accompanied by robust improvements in glycemic control and insulin sensitivity, resulting in attenuation of glucotoxicity and lipotoxicity, both of which contribute to endothelial dysfunction and myocardial injury. Additional benefits include reductions in blood pressure, favorable modulation of lipid profiles, and suppression of systemic inflammatory markers, alongside improvements in endothelial function and vascular stiffness.

Pharmacologically, dual GLP-1/GIP receptor agonists are engineered to provide balanced receptor activation, allowing superior metabolic efficacy compared with single GLP-1 receptor agonists. Clinical trial data indicate cardiovascular safety and improvements in surrogate cardiovascular endpoints, with reductions in major cardiometabolic risk factors comparable to those achieved with established incretin therapies. However, definitive evidence of incremental cardiovascular outcome benefits remains limited.

## Introduction and background

Cardiovascular diseases remain the leading cause of mortality worldwide and continue to impose a substantial global health burden despite advances in prevention and treatment. Importantly, a significant residual cardiovascular risk persists even among patients who achieve recommended targets for glycemic control, lipid levels, and blood pressure, highlighting the limitations of current therapeutic strategies. This unmet need is particularly evident in individuals with type 2 diabetes mellitus, in whom cardiovascular disease represents the primary cause of morbidity and mortality. These conditions share overlapping pathophysiological mechanisms and risk factors, underscoring the importance of integrated approaches that address the broader cardiometabolic continuum rather than isolated parameters [1].

Obesity and type 2 diabetes mellitus are major drivers of this continuum, contributing to atherosclerosis and heart failure through chronic inflammation, adipose tissue dysfunction, and adverse metabolic signaling that affect vascular and myocardial function [2,3]. These interconnected mechanisms promote endothelial dysfunction, vascular remodeling, and progressive cardiovascular injury.

Incretin-based therapies have emerged as an important strategy for cardiovascular risk reduction. Glucagon-like peptide-1 (GLP-1) receptor agonists have demonstrated definitive reductions in major adverse cardiovascular events, including myocardial infarction, stroke, and cardiovascular mortality, in large randomized trials [2,4]. These benefits extend beyond glycemic control and involve weight reduction, blood pressure lowering, lipid improvement, and attenuation of inflammatory and oxidative pathways [5].

More recently, dual GLP-1 and glucose-dependent insulinotropic polypeptide (GIP) receptor agonists have been introduced as a novel therapeutic class with promising cardiometabolic effects. Agents such as tirzepatide have shown substantial improvements in glycated hemoglobin, body weight, and lipid profiles in patients with type 2 diabetes mellitus and obesity. Trials such as SURPASS-4 and SURMOUNT-1 demonstrate favorable effects on cardiometabolic risk factors; however, definitive evidence of incremental reductions in hard cardiovascular outcomes remains under investigation [3,6,7].

The objective of this review is to examine the emerging role of dual GLP-1 and GIP receptor agonists in cardiovascular prevention, integrating current evidence on their mechanisms, metabolic effects, and clinical implications in individuals with obesity and type 2 diabetes mellitus.

## Review

Methods

This narrative review was conducted using a structured methodological framework to synthesize contemporary evidence on dual GLP-1 and GIP receptor agonists in cardiovascular prevention. The synthesis focused on major cardiovascular outcome domains, including major adverse cardiovascular events, heart failure outcomes, and cardiometabolic risk reduction in populations with obesity and type 2 diabetes mellitus.

A literature search was performed in PubMed, Scopus, and ScienceDirect for peer-reviewed articles published in English or Spanish between January 2020 and December 2025. The search combined Medical Subject Headings and free-text terms related to dual GLP-1/GIP receptor agonists, tirzepatide, cardiovascular outcomes, obesity, and type 2 diabetes mellitus using Boolean operators. The initial search identified 192 records; after duplicate removal and title-abstract screening, 64 full-text articles were assessed, and 27 studies were included in the final qualitative synthesis. Study selection was performed independently by two authors, with discrepancies resolved by consensus.

Eligible studies included randomized controlled trials, cardiovascular outcome trials, post hoc analyses, high-quality observational studies, systematic reviews, meta-analyses, and international guideline documents. Priority was given to studies reporting hard cardiovascular endpoints and predefined outcome measures. Preclinical studies without translational relevance and small uncontrolled series were excluded.

As this was a narrative review, no formal risk-of-bias tool was applied. Methodological quality was evaluated qualitatively based on study design, sample size, follow-up duration, outcome definitions, and overall validity. Artificial intelligence tools were used solely for structural organization and linguistic refinement; scientific interpretation and conclusions were determined exclusively by the authors.

Incretin physiology and cardiovascular relevance

GLP-1 is an incretin hormone secreted by intestinal L cells in response to nutrient intake. Its physiological actions are mediated through binding to GLP-1 receptors, which are widely expressed in multiple tissues, including the pancreas, heart, and vascular endothelium. Through receptor activation, GLP-1 enhances glucose-dependent insulin secretion, suppresses glucagon release, and delays gastric emptying, thereby contributing to postprandial glycemic regulation. Beyond its metabolic effects, activation of GLP-1 receptors exerts systemic actions that extend to cardiovascular tissues [6,8].

Accumulating clinical evidence has demonstrated that GLP-1 receptor agonists confer significant cardiovascular benefits that cannot be explained solely by improvements in glycemic control. Large cardiovascular outcome trials have shown reductions in myocardial infarction, stroke, and cardiovascular mortality among treated patients. These benefits are attributed to a combination of mechanisms, including enhancement of endothelial function, attenuation of inflammatory pathways, and direct cardioprotective effects at the myocardial and vascular levels. As a result, GLP-1 signaling has emerged as a key therapeutic pathway in cardiovascular risk reduction [2,4,5].

GIP represents a second incretin hormone with distinct but complementary physiological properties. It is secreted predominantly by K cells in the duodenum and proximal small intestine and stimulates insulin secretion in a glucose-dependent manner. Although its insulinotropic effect is markedly reduced in individuals with type 2 diabetes mellitus, growing evidence suggests that GIP retains important metabolic and vascular actions. Recent studies indicate that it may improve endothelial function and reduce the development of atherosclerotic plaque, highlighting a potential role in cardiovascular protection [6,9].

Historically, therapeutic interest in GIP was limited by the observation of impaired insulin responsiveness in type 2 diabetes mellitus. However, more recent findings have demonstrated that this apparent resistance is not absolute and may be reversible under certain conditions. This has led to renewed interest in GIP as a therapeutic target, particularly when its activation is combined with GLP-1 signaling, allowing exploitation of synergistic metabolic and vascular effects [6].

Dual activation of GLP-1 and GIP receptors offers complementary mechanisms of action that extend beyond those achieved with single incretin receptor agonism. Dual receptor agonists, such as tirzepatide, enhance insulin secretion and improve glycemic control while simultaneously exerting anti-inflammatory effects. In addition, these agents promote substantial weight loss and favorable changes in lipid profiles, which collectively contribute to a reduction in cardiovascular risk [6,10].

At a broader level, dual incretin receptor activation has shown promising effects on metabolic, vascular, and inflammatory pathways that are central to cardiovascular disease progression. Emerging evidence suggests that these combined mechanisms may translate into reductions in major adverse cardiovascular events and overall improvement in cardiovascular health. Such benefits appear to be mediated through integrated effects on metabolic homeostasis, suppression of chronic inflammation, and enhancement of vascular function, supporting the growing interest in dual GLP-1 and GIP receptor agonists as agents for cardiovascular prevention [2,11].

Pathophysiological basis for cardiovascular protection

Dual GLP-1 and GIP receptor agonists exert profound effects on body weight and adipose tissue distribution, which are central determinants of cardiometabolic risk. Tirzepatide, in particular, has demonstrated marked and sustained weight loss with a significant impact on the reduction of visceral adiposity. This effect is of particular relevance given the strong association between visceral fat accumulation and adverse cardiovascular outcomes. Evidence from the SURMOUNT-1 trial highlighted the efficacy of tirzepatide in inducing substantial body weight reduction in individuals with obesity, thereby contributing to meaningful improvements in cardiovascular risk profiles. By decreasing overall adiposity, tirzepatide favorably influences cardiometabolic health, an effect further supported by observed improvements in lipid parameters and reductions in inflammatory markers linked to cardiovascular disease [3,12,13].

Beyond its effects on body weight, dual incretin receptor agonism plays a critical role in improving glycemic control and insulin sensitivity. Enhanced insulin sensitivity represents a cornerstone in the management of type 2 diabetes mellitus and in the mitigation of cardiovascular risk. Tirzepatide has been shown to significantly lower glycated hemoglobin levels, reflecting robust improvements in glycemic control and reduced insulin resistance. Through these mechanisms, dual GLP-1/GIP receptor agonists effectively attenuate glucotoxicity and lipotoxicity, two interrelated processes that contribute to endothelial dysfunction, myocardial injury, and progressive cardiovascular damage. By lowering circulating glucose levels and improving metabolic efficiency, these agents help limit the downstream cardiovascular consequences of chronic metabolic dysregulation [3,14,15].

Tirzepatide also exerts beneficial effects on blood pressure and lipid metabolism, further reinforcing its cardioprotective profile. Clinical studies have consistently reported reductions in both systolic and diastolic blood pressure following treatment, effects that contribute directly to cardiovascular risk reduction. In parallel, favorable changes in lipid metabolism have been observed, including reductions in low-density lipoprotein cholesterol and triglyceride levels, which are well-established contributors to atherosclerotic cardiovascular disease [3,15].

In addition to these metabolic effects, dual GLP-1/GIP receptor agonists exert important anti-inflammatory and vascular actions. Tirzepatide has been associated with reductions in systemic inflammatory biomarkers, such as high-sensitivity C-reactive protein and intercellular adhesion molecule-1, both of which are closely linked to cardiovascular risk and atherosclerotic progression. Furthermore, improvements in endothelial function and reductions in vascular stiffness have been documented, highlighting beneficial effects on vascular integrity and compliance. These changes are essential for maintaining cardiovascular health and preventing the development and progression of atherosclerosis, and they further support the role of dual incretin receptor agonism as a comprehensive strategy for cardiovascular prevention [3,13,14].

Dual GLP-1/GIP receptor agonists: pharmacology

Dual GLP-1 and GIP receptor agonists have been specifically designed to achieve simultaneous activation of both incretin pathways, with tirzepatide representing the most advanced example of this therapeutic class. From a molecular perspective, these agents are engineered to bind and activate both the GLP-1 and GIP receptors in a coordinated manner with the aim of enhancing glucose-dependent insulin secretion, improving overall glycemic control, and inducing clinically meaningful weight loss. These combined effects directly address key determinants of cardiometabolic risk in patients with type 2 diabetes mellitus and obesity, thereby providing a mechanistic rationale for their potential role in cardiovascular prevention [6,10].

In contrast to single GLP-1 receptor agonists, dual incretin receptor agonists provide a more balanced and comprehensive receptor activation profile. This dual engagement is thought to amplify metabolic benefits by harnessing complementary signaling pathways, which may explain the superior efficacy observed with tirzepatide in terms of weight reduction and glycemic control when compared with GLP-1 receptor agonists alone. Such enhanced metabolic outcomes are of particular relevance, as improvements in body weight and glycemic parameters are closely linked to reductions in cardiovascular risk, suggesting that dual agonism may translate into more pronounced cardiovascular benefits [10,16].

From a pharmacokinetic standpoint, dual GLP-1/GIP receptor agonists such as tirzepatide are formulated for once-weekly subcutaneous administration, a feature that supports sustained receptor activation and improves treatment adherence. Their absorption, distribution, metabolism, and elimination profiles are optimized to maintain stable plasma concentrations over extended periods, making them well-suited for long-term management of chronic conditions such as type 2 diabetes mellitus and obesity. This prolonged pharmacological activity allows for consistent metabolic control, which is essential for long-term cardiovascular risk modification [6,17].

Pharmacodynamic analyses have further demonstrated a clear dose-response relationship with dual incretin receptor agonists. Higher doses of tirzepatide have been associated with progressively greater reductions in body weight and more pronounced improvements in glycemic control, underscoring the importance of dose optimization in clinical practice. Understanding this dose-dependent efficacy is critical for maximizing therapeutic benefits while minimizing adverse effects, and it provides clinicians with the flexibility to tailor treatment intensity according to individual patient needs and risk profiles [17].

Evidence from clinical trials

Dual GLP-1 and GIP receptor agonists have demonstrated favorable cardiometabolic outcomes in patients with type 2 diabetes mellitus, particularly through their effects on major cardiovascular risk factors. Agents such as tirzepatide have shown promising results in reducing the incidence of major adverse cardiovascular events alongside improvements in overall survival. A recent meta-analysis reported that both GLP-1 receptor agonists and dual GLP-1/GIP receptor agonists were associated with significant reductions in major adverse cardiovascular events, all-cause mortality, and cardiovascular mortality when compared with placebo, with no statistically significant differences observed between the two therapeutic classes. These findings suggest that dual agonists achieve cardiovascular risk reduction comparable to that of established GLP-1 receptor agonists in patients with type 2 diabetes mellitus [18].

When compared directly with GLP-1 receptor agonists, dual incretin receptor agonists appear to offer additional metabolic advantages that may further influence cardiovascular risk. Tirzepatide has consistently demonstrated superior efficacy in improving glycemic control and inducing weight loss, alongside broader reductions in cardiometabolic risk factors, when compared with traditional GLP-1 receptor agonists. These enhanced effects are attributed to the complementary mechanisms of dual receptor activation, which amplify metabolic benefits and may translate into incremental cardiovascular protection beyond that achieved with single receptor agonism [10,19].

Evidence from cardiovascular outcomes trials has been instrumental in defining the safety and potential benefits of tirzepatide. The SURPASS clinical trial program has provided key data indicating that tirzepatide does not increase the risk of major cardiovascular events in patients with type 2 diabetes mellitus and may confer benefits in populations at elevated cardiovascular risk. These findings support the cardiovascular safety of dual GLP-1/GIP receptor agonists and suggest a potential role in modifying long-term cardiovascular outcomes [20]. In parallel, improvements in surrogate cardiovascular endpoints have further strengthened this perspective. Reductions in blood pressure, favorable changes in lipid profiles, and decreases in inflammatory biomarkers have been consistently observed, pointing toward cardiovascular benefits that extend beyond glycemic control and are likely mediated by dual receptor agonism [5,10].

Although the majority of clinical evidence has focused on patients with type 2 diabetes mellitus, increasing attention has been directed toward the potential cardiovascular benefits of dual incretin receptor agonists in non-diabetic populations. In individuals with obesity, these agents may reduce cardiovascular risk primarily through substantial weight loss and improvements in metabolic parameters, even in the absence of overt diabetes. This has important implications for primary prevention, particularly in individuals with obesity or metabolic syndrome who are at heightened cardiovascular risk. Nevertheless, while the therapeutic potential of dual GLP-1/GIP receptor agonists in primary cardiovascular prevention is promising, dedicated cardiovascular outcomes trials in non-diabetic populations are still required to definitively establish their role in this setting [11,21].

Safety and tolerability

Dual GLP-1 and GIP receptor agonists are generally well-tolerated, although gastrointestinal adverse effects represent the most commonly reported safety concerns. Tirzepatide and other emerging agents, such as CT-388, have been associated with symptoms including nausea, vomiting, and diarrhea. These adverse effects are predominantly dose-dependent, with higher doses correlating with increased frequency and severity of gastrointestinal symptoms. In clinical studies involving tirzepatide, nausea and vomiting were reported more frequently at doses of 10 mg and 15 mg compared with the 5 mg dose, underscoring the importance of gradual dose escalation and individualized dose selection to enhance tolerability. Similarly, CT-388 has been described as generally well-tolerated, although mild gastrointestinal effects such as reduced appetite and nausea were observed during a four-week treatment period, indicating that gastrointestinal symptoms remain a class effect of dual incretin receptor agonism [22,23].

From a cardiovascular safety perspective, available evidence supports a neutral to potentially favorable safety profile for tirzepatide. A meta-analysis evaluating cardiovascular outcomes reported no increased risk of major adverse cardiovascular events, defined as a four-point composite endpoint, when tirzepatide was compared with control treatments. The hazard ratio was 0.80, with a 95% CI of 0.57-1.11, indicating no statistically significant elevation in cardiovascular risk. Importantly, this analysis included participants across a broad spectrum of baseline cardiovascular risk, and no significant effect modification was observed among different subgroups. These findings suggest that tirzepatide does not confer additional cardiovascular harm and may be considered a safe therapeutic option for patients with type 2 diabetes mellitus from a cardiovascular standpoint [20].

Despite these reassuring data, long-term safety information for dual GLP-1/GIP receptor agonists remains limited. Extended follow-up studies are required to fully characterize their safety profile over prolonged treatment durations. Ongoing concerns include the potential risk of hypoglycemia, particularly when used in combination with other glucose-lowering agents, as well as higher discontinuation rates observed at increased doses of tirzepatide. These issues highlight the need for continued pharmacovigilance and well-designed long-term clinical trials to better define optimal dosing strategies and ensure sustained safety in routine clinical practice [22].

Comparison with other cardioprotective therapies

GLP-1 receptor agonists have consistently demonstrated substantial cardiovascular benefits in patients with type 2 diabetes mellitus, including significant reductions in myocardial infarction, stroke, and cardiovascular mortality. These effects have been attributed not only to improved glycemic control but also to pleiotropic mechanisms such as anti-inflammatory actions, improvement of endothelial function, and protection against vascular injury, which collectively contribute to cardiovascular risk reduction. Within this context, dual GLP-1 and GIP receptor agonists, such as tirzepatide, have emerged as a potentially more potent therapeutic option. By combining activation of both incretin pathways, these agents have demonstrated superior efficacy in weight loss and glycemic control when compared with GLP-1 receptor agonists alone, effects that may translate into enhanced cardiovascular benefits through greater improvement of key cardiometabolic risk factors [2,4].

Sodium-glucose cotransporter 2 inhibitors represent another cornerstone of contemporary cardioprotective therapy, particularly due to their well-established role in reducing hospitalizations for heart failure and improving renal outcomes. Their cardiovascular benefits are largely independent of glucose-lowering and are thought to be mediated through hemodynamic effects, improved myocardial energetics, and modulation of neurohormonal pathways [24]. Evidence has shown that combining sodium-glucose cotransporter 2 inhibitors with GLP-1 receptor agonists provides incremental benefits, with significant reductions in heart failure-related outcomes and all-cause mortality compared with monotherapy. This combination strategy highlights the complementary nature of these drug classes in addressing different aspects of cardiovascular disease pathophysiology [25].

The potential for additive or synergistic effects among these therapies is of particular clinical interest. Studies evaluating the combined use of GLP-1 receptor agonists and sodium-glucose cotransporter 2 inhibitors have demonstrated a marked reduction in the risk of heart failure hospitalization, supporting the concept that targeting multiple cardiometabolic pathways can yield superior outcomes compared with single-agent approaches [26]. In this evolving therapeutic landscape, dual GLP-1/GIP receptor agonists may further enhance synergistic effects by simultaneously modulating glucose metabolism, body weight, inflammation, and vascular function. By influencing multiple interconnected mechanisms involved in cardiovascular disease progression, these agents hold promise as integral components of comprehensive cardiovascular prevention strategies [4].

Clinical implications and patient selection

Dual GLP-1 and GIP receptor agonists have emerged as a promising therapeutic option for selected patient populations, particularly individuals with type 2 diabetes mellitus who are at high cardiovascular risk. Agents such as tirzepatide appear especially suitable for patients who require more intensive cardiometabolic risk reduction and for those who have not achieved adequate glycemic control or weight loss with traditional GLP-1 receptor agonists alone. In addition, patients with obesity and related metabolic disorders may derive substantial benefit from the pronounced weight loss associated with dual incretin receptor agonism, which represents a key mechanism for reducing cardiovascular risk in this population [10,11].

The potential role of dual GLP-1/GIP receptor agonists spans both primary and secondary cardiovascular prevention, although their precise positioning within these contexts is still being defined. In secondary prevention, these agents may contribute to reducing the risk of major adverse cardiovascular events in patients with established cardiovascular disease by improving glycemic control, body weight, and other cardiometabolic parameters. At the same time, growing interest has focused on their potential application in primary prevention, particularly among high-risk individuals without overt cardiovascular disease but with obesity, type 2 diabetes mellitus, or metabolic syndrome. This area remains under active investigation, and further evidence is required to clarify the magnitude of benefit in such populations [5,27].

Integration of dual incretin receptor agonists into current clinical guidelines remains an evolving process. At present, guidelines strongly endorse GLP-1 receptor agonists for cardiovascular risk reduction in patients with type 2 diabetes mellitus, whereas the formal inclusion of dual GLP-1/GIP receptor agonists such as tirzepatide is still under evaluation [5]. Data from the SURPASS-CVOT trial suggest that although tirzepatide provides substantial benefits in glycemic control and weight reduction, its incremental cardiovascular advantage over established therapies may be modest. Consequently, future updates to clinical guidelines are likely to consider dual agonists for selected patient subgroups, pending the results of ongoing and future cardiovascular outcomes trials that further define their role in cardiovascular prevention [10,11].

## Conclusions

Dual GLP-1 and GIP receptor agonists represent a major advancement in incretin-based therapy by targeting multiple interconnected cardiometabolic pathways beyond glycemic control. Through combined effects on insulin secretion, weight reduction, lipid metabolism, blood pressure, inflammation, and endothelial function, these agents provide a strong mechanistic rationale for cardiovascular risk reduction.

Clinical evidence, particularly with tirzepatide, demonstrates superior weight loss and glycemic control compared with established GLP-1 receptor agonists, along with cardiovascular safety and favorable surrogate outcomes. However, definitive reductions in major adverse cardiovascular events, especially in primary prevention and non-diabetic populations, require confirmation through dedicated long-term cardiovascular outcomes trials to clarify their optimal role in prevention strategies.
